# Setting up an autonomic unit within the neurological department: a position statement of the European Federation of Autonomic Societies

**DOI:** 10.1007/s10286-026-01187-3

**Published:** 2026-02-10

**Authors:** Mario Habek, Alessandra Fanciulli, Anne Pavy-Le Traon, Walter Struhal, Magdalena Krbot Skorić, Luka Crnošija, Ivan Adamec, Marco Bozzali, Cristian Falup-Pecurariu, Jens Jordan, Evert Kaal, Gregor K. Wenning, Giovanna Calandra Buonaura, Giacomo Chiaro, Pietro Cortelli, Pietro Guaraldi, Max J. Hilz, Valeria Iodice, Astrid Juhl Terkelsen, Anita Kamondi, Marc Stefan Dawid Milner, Diogo Reis Carneiro, Camilla Rocchi, Isabel Rocha, Łukasz Rzepiński, Iva Stanković, Beatriz Tijero, Roland D. Thijs

**Affiliations:** 1 Department of Neurology, University Hospital Center Zagreb, Referral Center for Autonomic Nervous System Disorders, Kišpatićeva 12, 10000 Zagreb, Croatia; 2 School of Medicine, University of Zagreb, Zagreb, Croatia; 3 Department of Neurology, Medical University of Innsbruck, Innsbruck, Austria; 4 Neurology Department, UMR 1214 INSERM, University Hospital of Toulouse and ToNIC, Toulouse, France; 5 Department of Neurology, Karl Landsteiner University of Health Sciences, University Hospital Tulln, Tulln, Austria; 6 Faculty of Electrical Engineering and Computing, University of Zagreb, Zagreb, Croatia; 7 Department of Neuroscience Rita Levi Montalcini, University of Torino, Turin, Italy; 8 Department of Neurology, County Clinic Hospital, Transilvania University Brasov, Brasov, Romania; 9 Institute of Aerospace Medicine, German Aerospace Center (DLR), Cologne, Germany; 10 Medical Faculty, University of Cologne, Cologne, Germany; 11 Department of Neurology, Maasstad Ziekenhuis, Rotterdam, Netherlands; 12 IRCCS Istituto Delle Scienze Neurologiche Di Bologna, Bologna, Italy; 13 National Autonomic Centre, The National Hospital for Neurology & Neurosurgery, Queen Square, University College London Hospital NHS Foundation Trust, London, UK; 14 Alma Mater Studiorum – Università Di Bologna, Bologna, Italy; 15 University Erlangen-Nuremberg, Erlangen, Germany; 16 Icahn School of Medicine at Mount Sinai, New York, NY USA; 17 Department of Brain Repair and Rehabilitation, University College London Queen Square Institute of Neurology, London, UK; 18 Department of Neurology, Aarhus University Hospital, Aarhus, Denmark; 19 Department of Clinical Medicine, Aarhus University, Aarhus, Denmark; 20 Department of Neurology and Department of Semmelweis University, Semmelweis University, Budapest, Hungary; 21 Autonomic Nervous System Unit CIMES, School of Medicine, University of Málaga, Málaga, Spain; 22 Neurology Department, ULS Coimbra, Coimbra, Portugal; 23 Faculty of Medicine, University of Coimbra, Coimbra, Portugal; 24 Neurology Unit, Policlinico Tor Vergata, Rome, Italy; 25 Cardiovascular Autonomic Function Lab, Lisbon School of Medicine and CCUL@Rise, Universidade de Lisboa,, Lisbon, Portugal; 26 Department of Neurology, 10, Military Research Hospital and Polyclinic, Bydgoszcz, Poland; 27 Department of Clinical Medicine, Faculty of Medicine, University of Science and Technology, Bydgoszcz, Poland; 28 Faculty of Medicine, Neurology Clinic, University Clinical Center of Serbia, University of Belgrade, Belgrade, Serbia; 29 Neurodegenerative Diseases Group Biocruces Bizkaia Health Research Institute; Cruces University Hospital; CIBERNED, Barakaldo, Spain; 30 Stichting Epilepsie Instellingen Nederland (SEIN), 2103 SW Heemstede, Netherlands; 31 Department of Neurology, Leiden University Medical Centre (LUMC), Leiden, Netherlands; 32 UCL Queen Square Institute of Neurology, London, UK

**Keywords:** Autonomic nervous system, Unit, Europe, Diagnostics, European Federation of Autonomic Societies

## Abstract

**Purpose:**

The growing recognition of autonomic nervous system (ANS) dysfunction across a broad spectrum of neurological and systemic diseases calls for structured, multidisciplinary care. This position statement by the European Federation of Autonomic Societies aims to provide guidance on establishing an autonomic unit within a neurology department.

**Methods:**

A Task Force appointed by the European Federation of Autonomic Societies board in October 2022 proposed staffing and equipment requirements for autonomic units, based on a literature review, survey data from ANS laboratories, and existing diagnostic guidelines. These propositions were integrated into the initial 25 questions and refined using a modified Delphi method to achieve expert consensus. Consensus was predefined as ≥ 80% agreement. Statements that did not reach consensus were revised and reassessed in subsequent rounds.

**Results:**

After three rounds of Delphi surveys, expert consensus was achieved on the minimal and optimal requirements for autonomic unit personnel, technical equipment, and the availability of multidisciplinary care.

**Conclusion:**

This European Federation of Autonomic Societies position statement presents practical, expert-based recommendations to promote the development of autonomic units across various healthcare systems. By establishing minimum and optimal standards for staffing, equipment, and interdisciplinary collaboration, the document offers a clear framework for standardizing clinical care and supporting collaborative research. This statement serves as a foundational resource for clinicians, administrators, and policymakers committed to strengthening the ANS care infrastructure.

## Introduction

Advances in medicine and the growth of various subspecialties have emphasized the necessity for a multidisciplinary approach to managing complex diseases. Several examples demonstrate how creating multidisciplinary specialty units for different neurological conditions has significantly improved healthcare delivery for affected patients. A notable example is the development of stroke units. A recent Cochrane network meta-analysis showed that patients treated in specialized stroke units tend to have better outcomes, regardless of age, sex, initial stroke severity, or stroke type [1]. Similar findings have been observed in chronic neurological conditions such as multiple sclerosis (MS) [2], epilepsy [3], and amyotrophic lateral sclerosis [4]. Studies are also ongoing to appraise the prognostic gain of multidisciplinary healthcare provision models based on centers of excellence for disorders such as multiple system atrophy (MSA) that entail a combination of autonomic, motor, and other non-motor symptoms [5, 6].

Autonomic nervous system (ANS) disorders are prevalent, affecting an estimated 70 million people worldwide [7]. These disorders range from common conditions like vasovagal syncope and postural orthostatic tachycardia syndrome to rare diseases such as pure autonomic failure, MSA, hereditary sensory and autonomic neuropathies, primary amyloidosis, and hereditary ATTR amyloidosis (ATTRv) in non-endemic areas. Autonomic dysfunction also occurs in various neurological conditions, including stroke, MS, epilepsy, neurodegenerative and movement disorders, neuromuscular disorders, and many other medical diseases, highlighting the need for multidisciplinary healthcare. The impact of ANS disorders became especially clear during the COVID-19 pandemic, which saw a significant rise in persistent autonomic symptoms among those with post-COVID conditions, requiring specialized ANS care [8, 9] and adjustments to the testing protocols to operate safely during the pandemic [10, 11].

The disparity in ANS care across Europe is a significant concern. A joint effort by the European Academy of Neurology (EAN) and the European Federation of Autonomic Societies (EFAS) identified 84 neurology-led ANS laboratories in 22 European countries, with notably fewer labs in southern, eastern, and greater European regions [12]. The recent COVID-19 pandemic impacted the autonomic education and research [13]. More importantly, neurology residents and clinicians across Europe report uncertainties and unmet educational needs in various areas of autonomic medicine [14]. This poses a major obstacle to providing adequate healthcare for individuals with ANS disorders, particularly in underserved regions. The impact of knowledge gap is best studied in the field of syncope, the most common ANS symptom. Absence of care standards results in the widespread inappropriate use of multiple tests with only low diagnostic yield. Structured care pathways for syncope increases the diagnostic accuracy while lowering costs [15, 16]. Autonomic testing plays a critical role in the evaluation of complex cases [15, 16]. The EFAS, the EAN, the American Autonomic Society (AAS), the International Federation of Clinical Neurophysiology (IFCN), and the International Neuro-Urology Society (INUS) have issued consensus statements to define specific autonomic disorders and standardize their clinical assessment [17–21]. While these guidelines are helpful for diagnosing and treating ANS disorders, their successful implementation requires a comprehensive plan to establish new autonomic units or upgrade existing equipment and improve quality standards within current neurological autonomic centers. We therefore set out to delineate the requirements for an autonomic unit within the neurological department. By doing so, we aim to promote the development of new autonomic units in underserved areas and to support healthcare policy decisions related to the reimbursement for the diagnosis and treatment of ANS disorders.

## Methodology

In October 2022, the EFAS board established a Task Force (MH, AF, RT, MKS) to define the minimum and optimal staff and equipment necessary for establishing autonomic units within the neurology department. The framework of the EHRA recommendations for syncope care [22] served as the starting point, and this document aims to build upon it based on the necessities of additional care needs that come along with ANS disorders in neurological clinical practice.

The Task Force proposed staffing and equipment requirements for these specialized autonomic units based on a review of existing literature, metrics from the recent ANS laboratories survey [12], and guidelines or position statements for diagnosing ANS disorders [17–21]. We included this in the initial 25 questions of a modified Delphi process to reach consensus on the wording and strength of the recommendations, thus reducing bias that can arise from group dynamics. The modified Delphi method features anonymous voting, facilitated discussions, group feedback, and statistical analysis of responses [23].

All authors of the manuscript took part in the Delphi survey. They were chosen based on predefined criteria that required participants to be:

1. Clinicians or researchers with expertise in autonomic disorders
2. Actively working in or leading a neurology-driven autonomic laboratory
3. Regularly conducting and interpreting standardized ANS tests

These experts were selected from the European Network of Neurological Autonomic Laboratories, which was established through previous EAN/EFAS collaborative efforts. Participation was limited to those who met the above requirements to ensure that the consensus represented the views of experienced clinicians and researchers with practical laboratory experience.

The online Delphi survey was structured to include three rounds. Participants received summaries of the recommendations and an overview of the relevant literature for each question. The survey was conducted using Google Forms and distributed via personalized email links. All responses were anonymized throughout the process. Participants were asked to rate their level of agreement with each statement using these options and their definitions:

1. Strongly agree—I fully support this statement and believe it must be included as written.
2. Agree—I generally support this statement and consider it accurate and useful.
3. Neither agree nor disagree—I neither support nor oppose this statement; further clarification may help.
4. Disagree—I have reservations and would not support including this statement.
5. Strongly disagree—I believe this statement is clearly incorrect and should not be included.

The additional comments were available for the given response. For this Delphi survey, the consensus threshold was set beforehand at 80% of responses being “strongly agree” or “agree.” Recommendations that did not reach the consensus threshold were revised based on feedback and underwent a second or third Delphi round if needed.

## Results of the modified Delphi process

The first round of the Delphi survey, with its initial 25 questions, was conducted in June 2024 and involved 22 respondents. Participants discussed the results of Delphi round 1 on June 30, 2024, leading to the creation of a revised version of Table 1 for the Delphi round 2 survey, which included 13 questions and took place from August 2024 to October 2024, with 26 respondents. After the second round, two questions did not reach the 80% consensus, requiring a third round. The third round of the Delphi survey ran from January 2025 to March 2025, with 23 participants. Following three rounds, only one question failed to meet the required consensus level. After completing the Delphi process, the EFAS Board and the EFAS Council approved the final document during the European Academy of Neurology meeting in Helsinki on June 23, 2025.

**Table 1:**
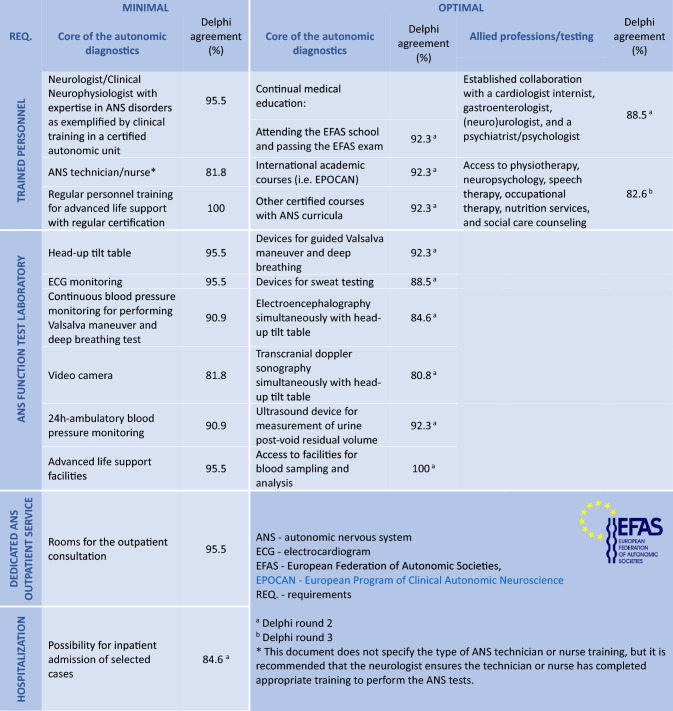
The requirements for the core of the autonomic diagnostics and allied professions/testing of the ANS unit

### Definition of an autonomic unit

The autonomic unit is a facility offering standardized multidisciplinary care for the diagnosis and treatment of ANS disorders. A recognized ANS center is defined as a neurology-driven autonomic laboratory that performs standardized ANS testing, adheres to established international guidelines (EFAS, EAN, AAS, IFCN), and has documented clinical activity in autonomic testing [17–21]. These centers also have the appropriate equipment and qualified personnel specializing in autonomic disorders, as outlined by EFAS criteria.

The objectives of neurological autonomic units include:

1. Standardizing healthcare provision for individuals with ANS disorders
2. Enabling screening and early, accurate diagnosis of ANS disorders
3. Offering appropriate diagnostic workup for suspected autonomic disturbances, including the exclusion of mimics
4. Offering individualized counseling and treatment for individuals diagnosed with ANS disorders
5. Early detection for initiating disease-modifying treatment or changing treatment in specific ANS disorders
6. Clinical autonomic research and contributions to international registries for common and rare ANS disorders
7. Healthcare planning (reducing disease burden and optimizing healthcare costs)
8. Clinical autonomic education

## The EFAS position statement on requirements for the autonomic unit

**Fig. 1:**
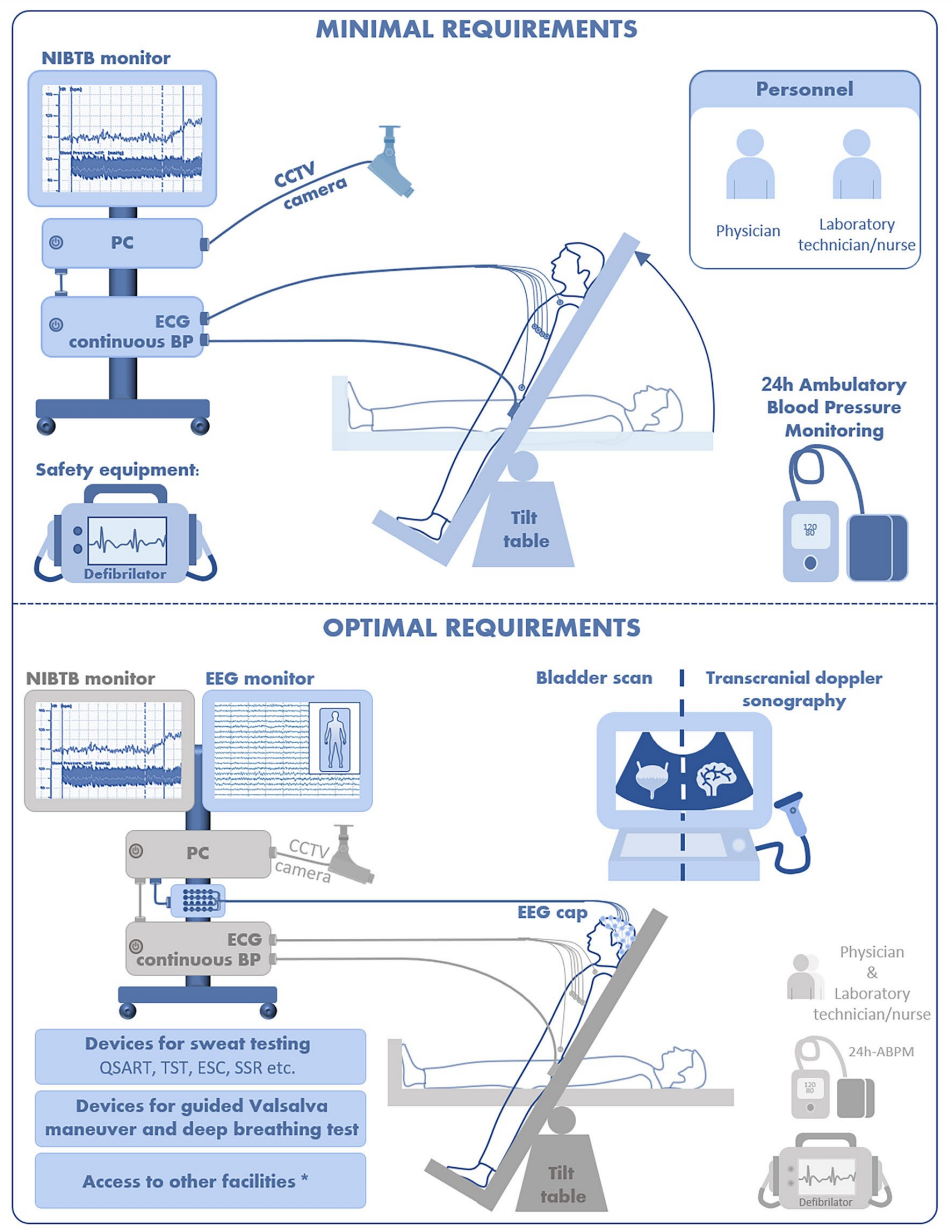
Schematic representation of the autonomic unit. The upper panel shows minimal requirements, and the lower panel shows optimal requirements, which are highlighted in blue. *Access to facilities for blood sampling and analysis, electroneurography, skin biopsy, neuro-urology, gastric and bowel motility, nuclear medicine, and radiology. *NIBTB *noninvasive beat to beat, *PC *personal computer, *CCTV *closed-circuit television, *ECG *electrocardiogram, *BP *blood pressure, *EEG *electroencephalography, *ABPM *ambulatory blood pressure monitoring, *QSART *quantitative sudomotor axon reflex test, *TST *thermoregulatory sweat testing, *SSR *sympathetic skin response, *ESC *electrochemical skin conductance

This position paper defines the core of autonomic diagnostics along with allied professions/testing that enable a more comprehensive evaluation of the ANS. A consensus was reached to divide the core of autonomic diagnostics into minimal and optimal requirements (Delphi round 2, agreement 88.5%). Table 1 lists all the minimal and optimal requirements that reached the preset consensus level (> 80%) through the modified Delphi process. The experts’ consensus level and the Delphi round in which it was reached are noted item by item. After three rounds of modified Delphi process only “pupillometry” as a part of the optimal requirements did not reach the required consensus level. This item is discussed in the “Pupillometry” section. To help visualize the required elements of an ANS unit, see the schematic diagram in Fig. 1.

## The rationale and considerations for ANS unit requirements

This section explains the rationale and some additional considerations for the requirements listed above. The items are organized by type (e.g., personnel, ANS laboratory, etc.), and by the level of requirement (i.e., minimal or optimal).

### Personnel requirements

#### Minimal

The neurological autonomic unit should be located within or affiliated with the neurology or neurophysiology department. The core team must include at least one physician with certified expertise in ANS disorders, such as ANS fellowships in a recognized ANS center, and an ANS technician or nurse. To promote effective transfer of clinical expertise, technical knowledge, and smooth operation of the ANS unit, it is highly recommended that at least one physician resident is a permanent staff member.

The core team acts as a contact point for referring physicians and patients. Nurses and technicians with specialized skills in ANS disorders assist the patient in preparing for testing and may also perform ANS tests under physician supervision, following internal protocols and standard operating procedures. The Task Force committee does not specify the training needed for ANS technicians and nurses but recommends that the neurologist ensure these professionals receive appropriate training to understand and perform ANS tests.

#### Optimal

##### Continual medical education

This item requires special attention. A survey involving over 220 neurology residents and consultants with varying experience from 33 European countries provides a comprehensive overview of the current state of autonomic neurology in clinical practice, while also underscoring the need for focused education on ANS disorders and outlining preferred educational formats [14]. The Task Force suggested two European educational ANS programs: the EFAS school and the European Program of Clinical Autonomic Neuroscience (EPOCAN). EFAS schools are held annually during the EFAS congress. The Task Force recommends attending at least two EFAS schools followed by the EFAS exam as one way to formalize educational requirements. Another option is to enroll in an international university course—EPOCAN. This program trains participants on how to assess the ANS using both basic and advanced diagnostic methods, and how to apply specific treatments based on a pathophysiological understanding of the disease mechanisms. Both options are recommended for clinicians entering the field and for establishing an autonomic laboratory, as well as for continuing medical education for those already practicing in autonomic medicine. The EFAS school offers structured, foundational training suitable for new practitioners, while also providing updated content valuable for experienced clinicians. The EPOCAN program delivers comprehensive, advanced education ideal for developing expertise when setting up a new laboratory and for further professional growth. Other certified courses with ANS curricula present additional educational options. For example, high-quality learning opportunities are also available through the American Academy of Neurology (AAN), the AAS, and Mayo Clinic. These courses and workshops offer both foundational and advanced training in autonomic disorders and serve as important supplementary educational resources for both new and experienced autonomic practitioners, supporting the global standardization of autonomic medicine education.

##### Allied professions/testing

The core team of the ANS unit should ideally be able to establish collaborations with a cardiologist, internist, gastroenterologist, (neuro)urologist, and a psychiatrist or psychologist knowledgeable about ANS disorders for shared care of complex cases with multidisciplinary needs on an “as needed” basis. Access to physiotherapy, neuropsychology, speech therapy, occupational therapy, nutrition services, and social care counseling is also preferred to provide optimal ANS care. Some examples supporting these collaborations include:

- Gastroenterologist—manages patients with disorders involving gut–brain interactions (such as irritable bowel syndrome and cyclic vomiting syndrome) or gastrointestinal motility issues (like gastroparesis and colonic inertia). Similar to the field of ANS neurology, the emerging field of neurogastroenterology has been growing in popularity since the beginning of this century. A recent paper advocates for increased efforts in this area and the development of interdisciplinary neurogastroenterology units [24].
- (Neuro)urologist—lower urinary tract (LUT) dysfunction is very common in many neurological disorders [25]. In specific cases like MSA, LUT dysfunction may be an early prodromal sign of the disease [26], opening opportunities for future research on early interventions.
- Cardiologist—a considerable number of patients eligible for ANS testing share much of the clinical picture with those seen by cardiologists. The role of cardiologists in managing patients with autonomic dysfunction has long been acknowledged, as evidenced by the fact that cardiologists are also members of autonomic societies and actively participate in defining and managing these conditions [18].
- Neuropsychologists and psychotherapists—probably the biggest use case for this collaboration lies in the management of patients with suspected psychogenic pseudosyncope and other types of functional transient loss of consciousness. Moreover, there are some indications that it may also be beneficial in some cases of patients with vasovagal syncope (VVS) [27, 28] or even postural orthostatic tachycardia syndrome (POTS) [29].

Therefore, actively pursuing collaborations and developing an interdisciplinary community of healthcare professionals aware of the field of ANS would surely lead to improved patient recognition and care, while also providing an exciting research opportunity in this relatively new area of medicine.

### ANS function test laboratory requirements

#### Minimal

##### Head-up tilt table

The head-up tilt table test (HUTT) was introduced into clinical practice in the 1970s, and since then, it has been routinely used in ANS laboratories [30]. HUTT can help identify the hemodynamic factors behind orthostatic intolerance and transient loss of consciousness [31] and is a core part of the ANS function test lab. The Task Force endorses the EFAS/EAN/AAS guidelines for HUTT indications and protocols [20]. Several factors should be considered when choosing the right head-up tilt table. The table must have a platform to support the patient’s feet in the tilted position, preferably with an adjustable or padded backrest to reduce patient discomfort. It must also be strong enough to safely support the patient’s weight. If an armrest is included, it should be height-adjustable to accommodate children during testing. The table should be wide enough to comfortably accommodate overweight patients. Safety belts are essential to prevent falls; they should include at least one belt across the chest and another above the knees, without constricting the legs [31]. Another key consideration is the tilting and reclining speed of the table. Motorized tilt tables vary in inclination rates, and some are manually adjusted. The effect of tilting speed has not been thoroughly studied [31]. However, speed may be crucial for re-establishing consciousness during head-up tilt-induced syncope. Therefore, we recommend that the tilt-back from 70° to horizontal should take less than 15 s [20]. Finally, the room housing the tilt table should be spacious enough to allow easy access from both sides in case of resuscitation. The room should keep a consistent ambient temperature of 22–24 °C, with controlled humidity, since both temperature and humidity can influence autonomic test results and patient comfort.

##### Electrocardiogram (ECG)

At least one ECG channel is necessary to detect signal artifacts, arrhythmias, and to determine their underlying mechanism (e.g., intermittent AV block) [20]. Most newer-generation devices for ANS testing use a 12-channel ECG. Raw ECG data, with the optimal sampling frequency needed to ensure adequate precision of R–R intervals, are essential for calculating heart rate variability at rest, during deep paced breathing, and other cardiovagal indices such as the Valsalva ratio.

##### Continuous blood pressure monitoring for performing Valsalva maneuver and deep breathing test

The use of a standard intermittent arm cuff alone is insufficient for autonomic testing, as it cannot capture the rapid, beat-to-beat blood pressure fluctuations required to assess baroreflex function, orthostatic responses, or transient hypotensive episodes. Assessing rapid and short-lived changes in blood pressure (BP) requires continuous recording on a beat-by-beat basis [32]. This is essential for detecting brief transient BP drops in reflex syncope or immediate orthostatic hypotension, as well as for evaluating baroreflex responses during the Valsalva maneuver. Continuous BP recording practices started in the 1970s with the development of finger cuffs that used infrared photoplethysmography. The advanced technology for noninvasive, continuous monitoring of finger blood pressure [33, 34] has become a gold standard in assessing ANS function. Finger BP must always be calibrated against brachial sphygmomanometry at baseline, as finger BP can give inaccurate absolute BP readings. In patients with digital vasoconstriction, finger BP may be significantly lower than brachial BP or may be unrecordable altogether. In such cases, passive warming of the hand (preferably with a heating lamp) can be used, although artifacts may still occur [35].

Some devices utilize a method called the “vascular unloading technique” [36, 37]. This method functions with an inflatable finger cuff fitted with an infrared photoplethysmograph designed to measure blood volume in the finger artery beneath the cuff. Another option is a compact device that provides noninvasive cardiovascular monitoring through various measurements: beat-to-beat finger BP recording using the vascular unloading technique; indirect cardiac output measurement via impedance cardiography; high-resolution ECG; and brachial sphygmomanometry [37].

There are several devices available for continuous BP monitoring that can be used in ANS laboratories. This position statement does not endorse any particular device, as long as it meets the previously mentioned requirements and complies with the European CE marking strategy for medical devices.

Alternative cuffless, noninvasive methods to continuously trace BP are currently under evaluation but are not yet clinically viable [38, 39].

High-quality digital acquisition of physiological signals is essential for accurate autonomic testing. Autonomic laboratories should therefore use digital recording systems that allow real-time signal visualization and storage, with adequate sampling rates to reliably capture rapid cardiovascular and respiratory changes. In particular, beat-to-beat blood pressure and ECG signals typically require sampling frequencies of 200 Hz or higher to ensure precise detection of R waves, systolic waveforms, and other key physiological features. Appropriate digitization safeguards data fidelity, improves interpretability, and facilitates standardized analysis across centers.

##### Video camera

Adding video recording to HUTT offers many benefits (Table 2) [20, 22, 40]. The choice of video camera depends on the laboratory setup and available equipment. A camera can be mounted on the tilt table to move up and down with the patient while staying focused on the patient’s head and shoulders. Alternatively, a ceiling-mounted camera provides an overview of the entire tilt table. The first setup allows for better visualization of the patient’s eye and facial movements, while the second setup enables visualization of the patient’s full body.

**Table 2:** Benefits of adding video recording during HUTT

Repeatable and objective review of clinical signs during TLOC
Increased reliability of clinical observation
Timing of TLOC with respect to hemodynamic parameters
Support the diagnosis of psychogenic pseudosyncope (functional TLOC)
Post hoc analysis, in case the potential crucial moments were unobserved by the physician
Assessing semiology of TLOC
Biofeedback/professional education

*TLOC* transient loss of consciousness, *HUTT* head-up tilt table test

Adapted from [40]

Integrating video recordings with the results of noninvasive beat-to-beat monitoring is an important step, as the timing of asystole relative to the loss of consciousness has significant implications for pacemaker considerations in individuals with cardioinhibitory vasovagal syncope and ictal asystole [41–43]. One practical and cost-effective method for combining video footage with HUTT recordings is capturing screen video data from the noninvasive beat-to-beat monitoring device along with real-time patient video, then storing the combined images as a new video signal [40].

##### 24-h ambulatory blood pressure monitoring (ABPM)

The addition of 24-h ABPM to the previously mentioned equipment has several advantages, such as evaluating postprandial hypotension, exercise-induced hypotension, real-life hypotensive susceptibility, and nocturnal hypertension [44]. It can also be a very useful tool in screening for cardiovascular autonomic function [45]. The currently available 24-h ABPM devices are small units connected to the arm cuff via tubing, measuring blood pressure at various times throughout the 24-h period. They typically weigh less than 1 kg, measure approximately 7 × 2.5 × 9 cm, have multiple cuff sizes available, and can be used on either the right or left upper extremity. The device should offer an option for patients to record BP when symptomatic. The home-based setting may enable capturing events during various daily activities, such as sitting and standing (i.e., postural change), before and after food intake, during exercise, as well as during and after medication. Therefore, the ABPM interpretation should ideally be accompanied by a detailed activity and posture diary, as posture-related blood pressure changes are crucial for assessing orthostatic intolerance, postprandial hypotension, and other autonomic disorders. The data from 24-h ABPM can vary and should be tailored based on the test’s indication. The Task Force recommends using a 15-min interval for BP recordings during the day and 30-min intervals during sleep [46]. A clearly written, patient-directed set of instructions on how to use the device and how to prepare a detailed activity diary on the day of the examination is crucial to maximize the effectiveness of 24-h ABP, specifically to link symptoms, activities, and BP changes during episodes. The diary format can vary widely, but the minimum data should include questions about bedtime and the time the patient fell asleep (including sleep quality), nocturnal bathroom visits, wake-up time, medication details (including type and timing), meals, physical activities, and symptoms related to orthostatic hypotension [47].

The future of monitoring BP changes may lie in continuous, noninvasive hemodynamic monitoring using small wearable sensors incorporated into finger rings [48], bracelets, or watches. Yet the clinical usefulness of vital signs collected via commercial, non-medical smart watches remains unclear for ANS disorders and should not be used for clinical decision-making.

##### Advanced life support (ALS) facility and regular personnel training

The occurrence of complications during ANS function testing is very rare [20]. However, some cases have been reported where life support measures were needed because of induced cardioinhibitory syncope with prolonged asystole [49]. Adult ALS includes advanced interventions that follow basic life support, such as the use of an automated or semi-automated external defibrillator. In any case, the 2021 European Resuscitation Council Guidelines for ALS should be followed [50]. Every member of the core ANS unit team, whether a physician or other professional with prescribing authority, regardless of the setting (hospital or outpatient or private practice), should be trained and capable of providing ALS when needed. Technologists and nurses working in the autonomic unit should hold current Basic Life Support (BLS) certification, while ALS-trained practitioners (physicians or other qualified staff) must be available on-site during autonomic testing. This ensures patient safety while aligning with realistic and widely accepted training standards. If the ANS unit is located in a hospital, there must be direct access to intensive care unit (ICU) physicians who are well-trained in resuscitation and life support.

#### Optimal

##### Devices for the guided Valsalva maneuver and deep breathing

Fast and short-lived changes in blood pressure (BP) and heart rate (HR) can be assessed, for example, during Valsalva maneuvers, deep breathing tests, or the sustained handgrip test [15]. The cardiovascular response for each of these procedures depends not only on the function of the ANS but also on the magnitude and duration of the stimuli applied [51]. The mouth air pressure during the Valsalva maneuver affects the amplitude of the arterial pressure response [52], and the extent of HR changes increases with deeper breaths [53].

Devices for the guided Valsalva maneuver and deep breathing are not mandatory for performing these maneuvers; however, some form of guidance should be used. For example, a simple way to ensure the correct air pressure during the Valsalva maneuver is to instruct a patient to blow air through a mouthpiece connected to an analog manometer while watching and maintaining the proper pressure reading on it [13]. More advanced and arguably more patient-friendly solutions are also available [54]. For the hand grip test, subjects maintain the necessary grip strength while watching the sphygmomanometer, which displays the actual force exerted. During deep, slow breathing, keeping the breathing rate steady can be supported, for example, with vocal guidance [55]. To reduce the influence of differences in stimulus application on the results of autonomic tests, several devices are available on the market to control stimulus self-application during the tests [55, 56]. These devices improve test reproducibility and accuracy, allowing tracking of individual variability in response to the test.

##### Electroencephalography (EEG) simultaneously with head-up tilt table

One of the few methods to study brain function during syncope is electroencephalography (EEG) [51]. A tilt table, along with video and EEG recording, is proposed as a useful diagnostic test for patients experiencing unexplained loss of consciousness [20, 57]. If the decrease in cerebral blood flow is sufficiently prolonged, the loss of consciousness can be accompanied by tonic, clonic, or myoclonic movements, which may be mistaken for epileptic seizures. Research has shown that the occurrence of specific clinical signs during syncope depends on whether the EEG signal exhibits flattening [41]. Event recording with EEG is the only procedure to provide definitive proof of functional TLOC [58]. Available research data suggest that about 1–8% of patients referred to ANS laboratory for recurrent syncope testing might actually have psychogenic pseudosyncope [59]. Interestingly, some patients may have both confirmed vasovagal syncope and pseudosyncope, again highlighting the need for interdisciplinary care and possible interventions [59]. Furthermore, a recent study demonstrated that the tilt table video-EEG test is cost-effective in assessing refractory episodes of loss of consciousness that are atypical for epileptic seizures [60].

In conclusion, incorporating an EEG into the HUTT presents several benefits for evaluating certain patients. The panel recommends integrating this measure into the ANS function test laboratory on a case-by-case basis.

##### Transcranial Doppler sonography (TCD) simultaneously with head-up tilt table

TCD measurements can be helpful in assessing individuals with syncope, orthostatic intolerance (POTS), chronic autonomic failure, and afferent baroreflex failure. Available data suggests that in some cases, cerebral blood flow measured by TCD during HUTT can be reduced and linked to the presence of orthostatic intolerance symptoms, even when no significant BP or HR changes are detected at the same time [61].

TCD ultrasound offers quick, noninvasive, real-time assessment of cerebrovascular function. TCD can measure flow velocity in large arteries to evaluate relative flow changes, identify focal vascular stenosis, or detect embolic signals within these arteries. It can also assess the health of a specific vascular territory by measuring blood flow responses to changes in BP (cerebral autoregulation), variations in end-tidal CO2 (cerebral vasoreactivity), or cognitive and motor activation (neurovascular coupling or functional hyperemia) [62]. TCD requires substantial operator expertise to obtain reliable insonation windows and high-quality velocity recordings, particularly during head-up tilt testing [63].

##### Devices for sweat testing

Sweat disturbance often occurs in individuals with ANS disorders and can be linked to both central (e.g., stroke, MS, neurodegenerative syndromes) and peripheral causes (e.g., diabetic neuropathy and other small fiber neuropathies of various etiologies). Research indicates that combining sudomotor function tests with standard hemodynamic tests (such as HUTT, Valsalva, etc.) can help differentiate between MSA with predominant parkinsonism and Parkinson’s disease [64]. However, testing sudomotor function comes with several cautions: the choice of test depends on the clinical syndrome being investigated [65], some tests involve high equipment costs, some are technically challenging, and others show high variability along with limited sensitivity and specificity. Therefore, the panel has decided to classify sudomotor testing as an essential part of the ANS function test laboratory. Of note, in centers where autonomic neuropathy or small fiber neuropathy is frequently evaluated, sweat testing should be considered a component, even if it remains classified as an optimal requirement in the general framework. It should be noted that the panel does not express a preference regarding the method used.

The following sudomotor function tests are available:

- *Quantitative sudomotor axon reflex test (QSART*): the most commonly used test of sudomotor function [66]. This test assesses postganglionic sudomotor sympathetic function and identifies the length-dependent pattern typical of polyneuropathy. Its advantages include quantifiable results, good temporal resolution, and relatively low variability [66]. However, its limitations involve the high cost of equipment, technical complexity, very low spatial resolution due to testing only four small areas, and potential discomfort from iontophoresis for the patient [66].
- *Thermoregulatory sweat testing (TST)* involves applying an indicator powder to as much of the patient’s skin surface as possible and raising the core temperature by about 1 °C in a controlled setting. The powder changes color upon contact with sweat, helping identify areas of anhidrosis [65]. The main benefits of this test are that it assesses the entire front of the body and detects abnormalities (such as anhidrosis) regardless of the location of the issue (whether central or peripheral, pre- or postganglionic) [15]. However, the test is technically challenging; results are qualitative (though the percentage of surface anhidrosis can be calculated), and the procedure can be time-consuming and uncomfortable, which may cause patients to hesitate to undergo repeated testing [65].
- *Sympathetic skin response (SSR):* The physiological basis of SSR is the presence of a polysynaptic reflex triggered by arousal stimuli, which ultimately causes the activation of sweat glands through sympathetic activity [67]. The SSR can be recorded in response to electrical stimulation of the median and tibial nerves, mental stress, cough, inspiratory gasp, loud noise, and various other somatic or psychological stressors [68]. The test is easily performed on most standard electromyography equipment, but the results are highly variable, habituate quickly, and SSR is generally considered to have limited sensitivity and specificity [65].
- *Electrochemical skin conductance (ESC):* The electrochemical skin conductance measures the change in skin conductance that occurs when a nickel electrode contacts sweat chloride in the presence of a low-voltage direct current. This method is considered an indirect marker for small fiber neuropathy and can be useful as a screening tool rather than a definitive diagnostic test [69].
- *Silicone impressions*: The silicone impression method samples the size and quantity of individual sweat droplets triggered by iontophoresis and collected as impressions on the surface of a silicone elastomer material applied to a small skin area, which is then allowed to harden [70]. Artifacts from hair, air bubbles, and skin surface variations can appear in the optically analyzed images. A variation of this test, the quantitative direct and indirect testing of sudomotor function (QDIRT), analyzes high-resolution digital images of sweat droplet impressions [71].

##### Ultrasound device for measurement of urine post-void residual volume

Neurogenic lower urinary tract (LUT) dysfunction is often reported by people with neurological diseases [21]. Since the ANS plays a crucial role in controlling the neural functions of the LUT, its assessment is often needed for the autonomic specialist. A team approach involving neurologists, urologists, and primary care doctors is vital for effectively managing neurogenic LUT dysfunction [21]. However, assessing post-void residual volume is critical for diagnosing and differentiating LUT problems, as well as for management strategies like establishing voiding schedules, studying bladder biofeedback, and reducing urinary tract infections [72]. One key requirement for an ANS laboratory is the ability to measure post-void residual urine volume. These measurements are usually taken using ultrasound to estimate the amount of urine left in the bladder after urination, or via straight catheterization. Portable bladder ultrasound devices are mobile tools that use automated technology to digitally record bladder volume, including residual urine, and to produce three-dimensional images of the bladder [72].

##### Access to facilities for blood sampling and analysis

Access to other departments or hospital facilities for blood sampling and analysis (e.g., catecholamines, specific antibodies, etc.) should be considered when establishing an ANS function test laboratory, as they may be necessary for the diagnostic workup of some cases.

##### Pupillometry (consensus not reached)

The pupillometry was the only element of the optimal core of autonomic diagnostics that did not meet the preset limit across all three Delphi rounds. To the best of our knowledge, this method is generally missing from the current ANS laboratories’ inventory and, therefore, is not widely used in ANS practice. However, it has promising potential applications, such as measuring (para)sympathetic indices through analyzing pupillary motility [73].

The fluctuations in pupil size result from ANS activity. Pupillometry measures baseline pupil size and the rapid pupillary response using eye-tracking systems, and it evaluates the pupillary light reflex with automated pupillometers. This technique is becoming increasingly popular in research [74]. In recent years, infrared devices integrated into digital cameras have enabled the development of portable, user-friendly digital systems. These systems allow researchers to perform noninvasive, repeatable assessments of pupil size and light reactivity through an objective, accessible, and cost-effective approach [74]. The method is considered reliable for obtaining pupillary measurements at the bedside, particularly in ICUs [75].

### Dedicated ANS outpatient service requirements

#### Minimal

Besides the room(s) for formal ANS testing procedures, the neurological autonomic unit should include an outpatient service with consulting and examination rooms. In Europe, about 75% of neurology-driven ANS laboratories have an ANS outpatient clinic, with a median of 200 (IQR 100–360) outpatient visits annually [12]. The outpatient service can either be linked to the ANS function test laboratory or integrated within other neurological outpatient services, offering dedicated space and appointment slots for patients with ANS.

The availability of dedicated outpatient services for ANS disorders is crucial in maintaining quality of care and preventing adverse outcomes. This need was underscored during the COVID-19 pandemic, when an average 45% drop in ANS outpatient visits across European countries led to a significant decline in care quality and major adverse events due to missed care were reported in one out of every three autonomic centers [9].

### Hospitalization requirements

#### Minimal

Some patients may present with (sub)acute, severe, and sometimes life-threatening disturbances of ANS function, warranting urgent and thorough evaluation as well as targeted treatment (e.g., paraneoplastic autonomic neuropathy [76] or autoimmune autonomic ganglionopathy [77]). Prompt access to multidisciplinary management options is another important reason for hospitalizing certain patients. Currently, inpatient admissions are available in 91% of neurology-driven ANS laboratories, with a median of 20 (IQR 4–110) inpatient stays per year [12].

## Conclusion

With the increasing recognition of ANS disorders in various neurological and other medical conditions and considering the rising demand in the post-COVID-19 era, this initiative addresses current disparities in access to autonomic testing and calls for broader policy support and implementation. This position statement by the European Federation of Autonomic Societies provides practical recommendations to support the development of autonomic units, outlining minimal and optimal requirements for staffing, equipment, and interdisciplinary collaboration. Establishing standardized autonomic units across Europe is a crucial step toward a more holistic, high-quality patient-centered model of care, aligning directly with the EAN Brain Health Mission, which emphasizes early detection, prevention, and integrated care across the lifespan. Additionally, autonomic units foster cross-specialty collaboration and serve as hubs for research, education, and innovation. This document lays out the foundation for negotiations with local and healthcare policymakers.

We acknowledge that the current evidence directly linking specific autonomic diagnostic procedures to improved clinical outcomes remains limited. Most autonomic tests have not yet been evaluated in prospective, outcome-driven studies, and clinical practice often relies on physiological principles and expert experience rather than randomized controlled data. Consequently, a consensus-based methodology such as the modified Delphi process was essential for developing harmonized recommendations across diverse European autonomic laboratories. These consensus-derived requirements therefore represent a pragmatic framework for standardizing care while also highlighting critical gaps in the evidence base. Addressing these gaps through future research will be crucial for determining which autonomic assessments most meaningfully influence diagnosis, therapeutic decisions, and long-term patient outcomes, ultimately paving the way toward truly evidence-based autonomic medicine.

## Data Availability

The data supporting this study’s findings are available from the corresponding author upon reasonable request.
